# Influence of the Loading with Newly Green Silver Nanoparticles Synthesized Using *Equisetum sylvaticum* on the Antibacterial Activity and Surface Hardness of a Composite Resin

**DOI:** 10.3390/jfb14080402

**Published:** 2023-07-28

**Authors:** Ionuț Tărăboanță, Ana Flavia Burlec, Simona Stoleriu, Andreia Corciovă, Adrian Fifere, Denisa Batir-Marin, Monica Hăncianu, Cornelia Mircea, Irina Nica, Andra Claudia Tărăboanță-Gamen, Sorin Andrian

**Affiliations:** 1 Faculty of Dental Medicine, Grigore T. Popa University of Medicine and Pharmacy, 16 Universitatii Str., 700115 Iasi, Romania; ionut-taraboanta@umfiasi.ro (I.T.); irina.nica@umfiasi.ro (I.N.); andra-claudia.gamen@umfiasi.ro (A.C.T.-G.); sorin.andrian@umfiasi.ro (S.A.); 2 Faculty of Pharmacy, Grigore T. Popa University of Medicine and Pharmacy, 16 University Str., 700115 Iasi, Romania; ana-flavia.l.burlec@umfiasi.ro (A.F.B.); mhancianu@yahoo.com (M.H.); corneliamircea@yahoo.com (C.M.); 3 Centre of Advanced Research in Bionanoconjugates and Biopolymers Department, “Petru Poni” Institute of Macromolecular Chemistry, 41A Grigore Ghica Voda Alley, 700487 Iasi, Romania; fifere@icmpp.ro; 4 Faculty of Medicine and Pharmacy, Dunărea de Jos University, 800010 Galati, Romania; denisa.batir@ugal.ro

**Keywords:** AgNPs, *Equisetum sylvaticum*, *Streptococcus mutans*, microhybrid composite resin, Vickers hardness

## Abstract

The aim of the study was to evaluate the antibacterial activity and surface hardness of a light-activated microhybrid composite resin modified with green silver nanoparticles (AgNPs). AgNPs were synthesized using an *Equisetum sylvaticum* extract and characterized through different methods such as UV-Vis, EDX, and FTIR. The obtained AgNPs were mixed with a microhybrid composite resin (Herculite XRV, Kerr Corp., Orange, CA, USA) in different concentrations: 0% (group A-control); 0.5% (group B); 1% (group C); and 1.5% (group D). A total of 120 composite resin disk-shaped samples were obtained and divided into 4 groups (n = 30) according to AgNP concentration. Each group was then divided into 2 subgroups: subgroup 1—samples were not soaked in 0.01 M NaOH solution; and subgroup 2—samples were soaked in 0.01 M NaOH solution. The antibacterial activity against *Streptococcus mutans* was determined using a direct contact test. A digital electronic hardness tester was used to determine the composite resin’s Vickers surface hardness (VH). Statistical analysis was performed using the Mann–Whitney U and Kruskal–Wallis nonparametric tests with a confidence level of 95%. Groups C and D showed higher antibacterial activity against *S. mutans* when compared to the control group (*p* < 0.05). No significant differences were recorded between VH values (*p* > 0.05). The use of AgNPs synthesized from *Equisetum sylvaticum* as a composite resin filler in 1% wt. and 1.5% wt. reduced the activity of *Streptococcus mutans*. Soaking of the experimental composite resin decreased the antibacterial efficacy. The loading of a microhybrid composite resin with AgNPs in concentrations of 0.5% wt., 1% wt., and 1.5% wt. did not influence the surface hardness.

## 1. Introduction

The oral cavity is a conducive environment for development and multiplication of pathogenic microorganisms [1]. Some oral pathogens are associated with the development of carious or periodontal disease [2]. One of the common causes of direct restorations failure is the occurrence of secondary caries due to the adhesion of the bacterial biofilm to the tooth surfaces or to restorative materials [1]. In the absence of an adequate treatment, dental caries and periodontal diseases can evolve into systemic diseases such as infectious endocarditis or respiratory diseases [3,4]. Pathogenic microorganisms form the bacterial biofilm that attaches to dental surfaces, to the surrounding tissues, or to the surface of restorative materials [5]. Then it is important for dental materials used for direct restorations to have antimicrobial properties [1]. Some of the mostly used antibacterial components are: monomer MDPB (12-methacryloyloxydodecyl pyridinium bromide), antibiotics, silver nanoparticles (AgNPs), chlorhexidine digluconate (CHX), ursolic acid, or chitosan [1,4].

Silver, initially used in the form of silver nitrate (AgNO_3_) and later in combination with fluoride (AgF), has been used in oral care for a long time, becoming widespread in the 19th century as a component of dental amalgams. In the middle of the 20th century, when polymer resin appeared on the market, its popularity decreased [6]. In recent years, as a result of developments in the nanotechnology field, the use of silver in the form of nanoparticles smaller than 100 nm has been highlighted in various industries such as pharmaceuticals, healthcare, information technology, and cosmetics [7,8,9]. The estimates show continuous growth of the silver nanoparticle market in the following years [9]. The use of AgNPs as antimicrobial agents incorporated into the inorganic component of direct dental restorative materials has become very popular due to the prolonged antimicrobial effect, the reduction of biofilm colonization potential [10], and the reduced volatility [1]. The antimicrobial effect of AgNPs relies on the silver content (ranging between 10,000 and 15,000 silver atoms) and on increased surface/volume ratio [11]. A perfect explanation for AgNPs’s mechanism of action is not known, but due to their small size, they interact with the peptidoglycan cell wall, increasing membrane permeability, resulting in cell death [10]. According to Gudkov et al. [12], the antimicrobial efficacy of AgNPs is manifested by at least 5 mechanisms, such as the destruction of the cell wall and cytoplasmic components as a result of binding to the cell envelope, inactivation of respiratory chain dehydrogenases, inhibition of ATP synthesis and production of oxidative stress [13,14], exerting a genotoxic activity, blocking the potential of cell multiplication, and a photocatalytic effect [12]. At the same time, such nanoparticles block the multiplication potential of the bacterial DNA by condensing it [13,14] and disrupting the enzymatic activity by attacking thiol groups [15].

Previous studies have demonstrated that the use of AgNPs in direct dental restorative materials offers bacteriostatic or bactericidal effects on oral bacteria such as *Streptococcus mutans* or *Lactobacillus acidophilus* [16,17], increased biocompatibility [10], and reduced cytotoxicity and immunological response [12]. Synthesis of AgNPs can be achieved by chemical, physical, and biological methods [18]. At this time, the synthesis of AgNPs using plant extracts is preferred due to increased accessibility and improved biological properties [19]. The *Equisetum* genus from the *Equisetaceae* family, also known as “horsetail”, is found in Europe, North, Central, and South America and is used in the treatment of lung, kidney, or gastrointestinal ailments [20]. Species from this genus contain several bioactive compounds such as flavonoids, quercetin glycosides, phenolic acids, campesterol, phytosterols, alkaloids, and isofucosterol [21,22]. Nonetheless, a small number of studies that focus on species of the *Equisetum* genus can be found in the existing literature [19].

Consequently, the aim of this study was to assess the surface hardness and antibacterial efficacy against *S. mutans* of a microhybrid composite resin loaded with AgNPs obtained from an ethanolic extract of *Equisetum sylvaticum*. The null hypothesis was that there are no differences in hardness and antibacterial effect against *S. mutans* of the new experimental microhybrid composite resin when comparing to an unmodified composite material.

## 2. Materials and Methods

This study was performed in accordance with the Declaration of Helsinki and complied with all the rules imposed by the Ethics Commission of “Grigore T. Popa” University of Medicine and Pharmacy Iași (no. 291/10.04.2023).

**Figure 1:**
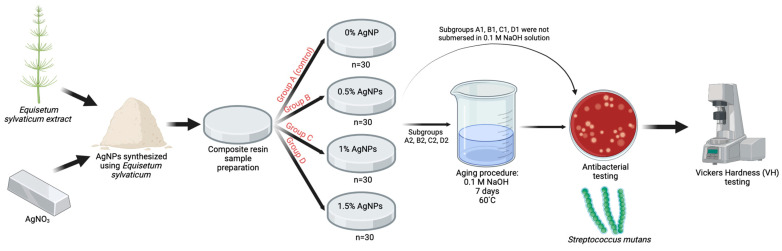
Schematic representation of the study protocol.

A schematic representation of the study protocol is shown in Figure 1.

### 2.1. Synthesis of AgNPs and Optimization and Characterization

Initially, the *Equisetum sylvaticum* extract was obtained. Ten grams of dried and finely crushed plant material were mixed with 100 mL of water in an ultrasonic bath at 30 °C for 30 min. Afterwards, the mixture was filtered through Whatman no. 1 filter paper. The extract was stored at 4 °C in a dark place until further use.

The extract was used as a source of reducing agent and AgNO_3_ as a precursor, the reaction being conducted at room temperature by magnetic stirring. For the optimization of synthesis, four parameters were taken into consideration: AgNO_3_ concentration, pH, extract:AgNO_3_ volume ratio, and reaction time. After establishing the reaction conditions, the colloidal solution containing AgNPs was centrifuged at 10,000 rpm for 30 min. To remove unwanted impurities, the obtained AgNPs were redispersed in water, centrifuged, and separated, with the operation being repeated twice. The purified AgNPs were dried and stored for further experiments.

To confirm the formation of AgNPs, the reaction mixture’s color change (extract: AgNO_3_) was monitored, and then the solution was examined using a UV-Vis spectrophotometer (Jasco V530, Jasco Inc., Tokyo, Japan) in the 400–500 nm range at various intervals.

Fourier transform infrared spectroscopy (FTIR) spectra were obtained by analyzing the pellet containing extract and AgNPs, respectively, in potassium bromide over the 4000–310 cm^−1^ spectral range using a Bruker Vertex 70 spectrophotometer (Bruker Corp. Berlin, Germany). Qualitative analysis of AgNPs was performed using a Quanta 200 environmental scanning electron microscope (ESEM) with energy dispersive X-ray spectroscopy (EDX). A Delsa Nano submicron particle size analyzer (Beckman Coulter, Brea, CA, USA) was used to measure the average diameter and the zeta potential value.

Transmission electron microscopy (TEM) studies were carried out with a Hitachi High-Tech HT7700 transmission electron microscope (Hitachi, Tokyo, Japan) that operated at a 100 kV accelerating voltage in high-contrast mode. The sample preparation methodology was as follows: a drop (10 μL) was placed on carbon-coated copper grids with 300-mesh size, and then the solvent was allowed to evaporate at room temperature.

### 2.2. Composite Resin Sample Preparation

To determine the sample size, G* Power software (Heinrich-Heine Universitat Dusseldorf, Dusseldorf, Germany) was used with an effect size set to 0.35, considered a medium effect according to Cohen’s classification, an alpha value of 0.05, and a power of 0.80. The results obtained indicate the use of a total of 96 samples.

For sample preparation, Herculite XRV microhybrid composite resin (Kerr Corp., Orange, CA, USA) was used. The used composite material is composed of a resin matrix based on BisGMA, TEGDMA, and UDMA monomers and barium/silicium fillers in 79% wt., with an average size of 0.6 µm. A Kern PCB high-precision scale (Kern&Sohn GmbH, Balingen, Germany) was used to weigh the materials. The synthesized AgNPs were incorporated into the composite material in different concentrations of 0% wt., 0.5% wt., 1% wt., and 1.5% wt. The incorporation of AgNPs was performed in sterile containers devoid of light, and for the homogenization process, a magnetic stirrer set at 200 rpm was used for 20 min.

A total of 120 disk-shaped samples of 2 mm height and 5 mm width were divided into 4 groups according to loading level of composite resin with the synthetized AgNPs: group A (n = 30)—0% AgNP load (control); group B (n = 30)—0.5% AgNP load; group C (n = 30)—1% AgNP load; and group D (n = 30)—1.5% AgNP load. Each group was further divided into 2 subgroups: subgroup 1—samples were not soaked in 0.01 M NaOH solution; subgroup 2—samples were soaked in 0.01 M NaOH solution. The samples were prepared at an ambient temperature of 23–25 °C, humidity conditions of 65–75%, and low lighting. To obtain the samples, the microhybrid composite resin was inserted into a cylindrical acrylic mold and covered on both sides with 2 celluloid strips and 2 glass plates. A constant pressure was applied with a 500 g weight for 30 s to remove air voids and obtain a smooth surface. The photoactivation of the material was performed for 40 s using a Bluephase 20i light-curing lamp (Ivoclar, Vivadent, Schaan, Lichtenstein) with a wavelength between 285 and 515 nm and a light intensity of 1200 mW/cm^2^. A Bluephase Meter II radiometer (Ivoclar, Vivadent, Schaan, Lichtenstein) was used to measure the light intensity for each light activation. The samples were finished and polished using a SofLex finishing and polishing system (Batch No. NC11346, 3M ESPE, St. Paul, MN, USA). The system consists of two spiral wheels (beige and white) made of thermoplastic elastomer impregnated with aluminum oxide particles. Finishing was performed for 1 min (30 s for each wheel) under a continuous water jet at a conventional speed of 20,000 rpm. Afterwards, the samples were sterilized in an autoclave (Dr. Mayer GmbH, Kempten, Germany) and stored in physiological serum at a constant temperature of 37 °C in an incubator (Biobase BJPXH30II, Biodusty, Shandong, China). Then, the samples were soaked for 7 days in a 0.1 M NaOH solution at a constant temperature of 60 °C, according to a protocol described by Prakki et al. [22]. The solution pH was 13, and it was checked every 24 h using a portable pH meter (Thermo Scientific Eutech pH 5+, Vernon Hills, IL, USA).

### 2.3. Antibacterial Analysis

The analysis of antimicrobial efficacy against *S. mutans* (ATCC^®^25175™) was performed using a direct contact test [23]. The bacterial suspension, with a content of 1.5 × 10^8^ bacteria/1 mL, was cultured in a brain–heart infusion (BHI) broth with a concentration of 0.5 McFarland. The McFarland suspension was subsequently diluted 10^3^ times to a final concentration of 1.5 × 10^5^ to reduce the number of bacterial colonies and facilitate their visual quantification. A 0.01 mL volume of bacterial suspension was applied to the surfaces of sterile composite resin discs, after which the samples were placed in test tubes containing 0.5 mL of BHI and incubated for 12 h in an incubator (Biobase BJPXH30II, Biodusty, Jinan, China) at 37 °C in a 5% CO_2_ atmosphere. Afterwards, 0.01 mL of liquid was collected from each culture medium and applied to a 5% sheep blood agar plate. After the incubation period, the colony-forming units (CFUs) were calculated using the following formula:

$$CFU=\frac{Number\;of\;colonies\times Dilution\;factor}{Volume\;of\;culture\;plate}$$

This assay was performed in triplicate.

### 2.4. Vickers Hardness Evaluation

To determine the Vickers surface hardness on both surfaces of the samples, a digital electronic hardness tester (Micro-Vickers Hardness System CV-400DMTM, CV Instruments Namicon, Otopeni, Romania) equipped with special indentation tips and a software program for measurement and evaluation were used. For each sample, 3 successive indentations were made on both sides of the composite resin discs, respecting a distance of 1 mm between them. A dwell time of 30 s and a load of 50 g was applied to the determination tip. The criteria upon which the indentations were accepted consisted of the existence of a uniform appearance, no irregularities, and sharp, diagonal edges. To determine the hardness, the diagonal of the indentation was measured, and the results were expressed in Vickers hardness numbers (VHN), calculated based on the formula D = 1854.4 × F/d^2^ (N/mm^2^), where D is the Vickers hardness value, F is the test force, and d is the length of the diagonal. The VHN values of each sample resulted from the mean of the 3 determinations.

### 2.5. Statistical Analysis

Statistical analysis was performed using SPSS 29.0 software (IBM SPSS Inc., Chicago, IL, USA). The Shapiro–Wilk test was used to test the normality of distribution and Levene’s test was used to assess the homogeneity of variances. The nonparametric statistical tests Mann-Whitney U and Kruskal–Wallis were used due to the heterogeneity of the variances and large dispersion of the data, with a significance level of 0.05.

## 3. Results

### 3.1. Optimization of AgNP Synthesis and Physico-Chemical Characterization

**Figure 2:**
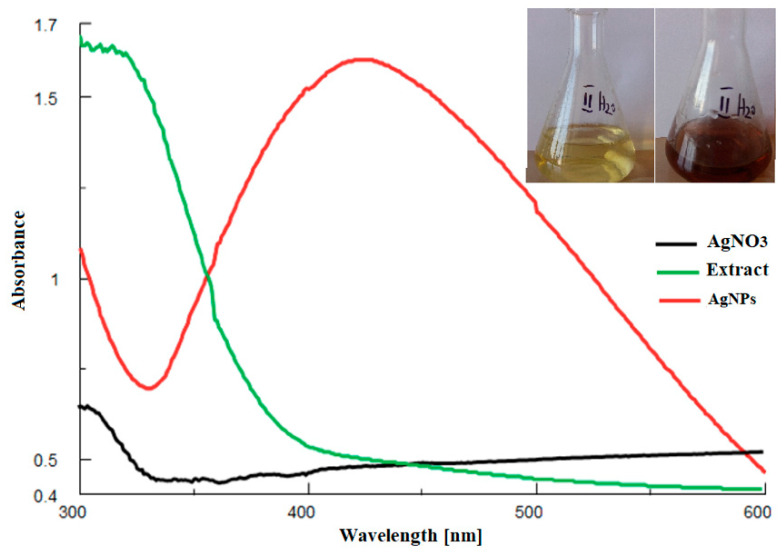
UV-Vis comparative spectra of the extract, AgNO_3_ solution, final mixture. Inset: color modification: initial vs. final.

Firstly, the synthesis was demonstrated by the color modification of the reaction mixture (extract:AgNO_3_) from yellow to dark brown in 300 min and confirmed by comparing the UV-Vis absorbance spectra of the extract, AgNO_3_ solution, and mixture (Figure 2). No absorbance peak was observed for the extract or AgNO_3_ in the 400–500 nm range, but a peak appeared at 440 nm for the mixture.

**Figure 3:**
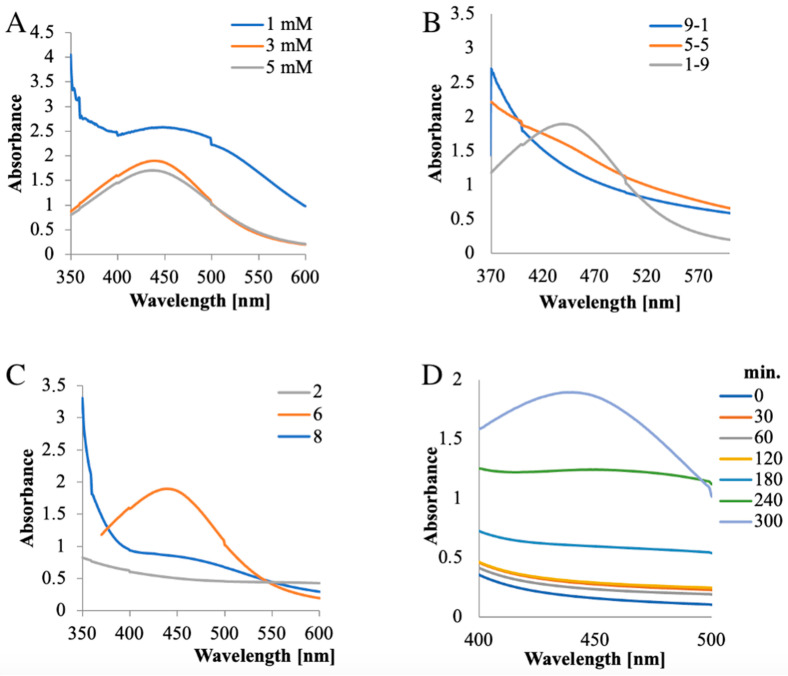
Optimization of parameters for AgNP synthesis: (**A**) AgNO_3_ concentration; (**B**) extract:AgNO_3_ volume ratio; (**C**) pH; (**D**) time.

In the process of optimization, the extract concentration was maintained constant (10 g%), but different AgNO_3_ concentrations were used (1 mM, 3 mM, and 5 mM) (Figure 3A). It was observed that, in all three cases, the AgNPs peak appeared, but its aspect was different: for 1 mM, the peak was large, while for 3 mM and 5 mM, the peak appeared sharper and without a significant increase in the absorbance value. Therefore, 3 mM AgNO_3_ was used for further studies.

Several extract:AgNO_3_ volume ratios (9:1, 5:5, and 1:9) were examined using constant extract (10 g%) and silver salt (3 mM) concentrations (Figure 3B). For the 9:1 and 5:5 extract:AgNO_3_ (*v*:*v*) ratios, no peak was observed, but the peak appeared at a 1:9 ratio; hence, this proportion was considered optimum for synthesis.

When optimizing the pH required for the reaction, it was found that, at pH 2, the synthesis was suppressed, while at pH values of 6 and 8, AgNPs were obtained. However, at pH 8, the peak was lower compared to that obtained at pH 6. Consequently, the last value was considered optimal for the synthesis (Figure 3C).

The optimal time required for synthesis was also examined. Initially, the reaction was slow; the peak appeared after 240 min and increased up to 300 min, after which there was no further significant increase, thus it can be considered that the synthesis was completed in 300 min (Figure 3D).

Therefore, the optimal conditions considered for the AgNP synthesis were: 3 mM AgNO_3_ concentration, 1:9 (*v*/*v*) extract:AgNO_3_ ratio, a pH of 6, and a 300 min synthesis time.

### 3.2. DLS Characterization, Zeta Potential, and EDX Analysis

**Figure 4:**
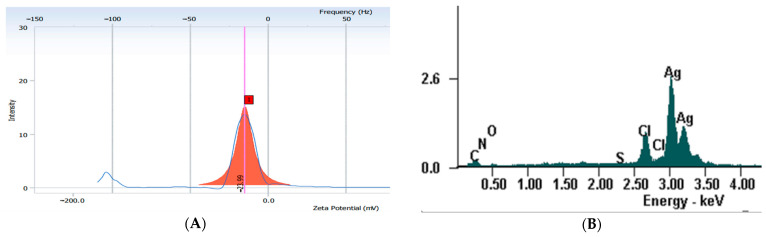
(**A**) Zeta potential; (**B**) EDX spectrum. 1—Zeta potential value.

The Zeta sizer showed an average hydrodynamic diameter of 136 nm with a polydispersity index of 1.116 for the prepared AgNPs. The obtained Zeta potential value was −23.99 (Figure 4A).

The EDX qualitative analysis of the AgNPs pointed out the characteristic peak for metallic silver at 3 keV (Figure 4B). The EDX quantitative analysis showed that the AgNPs contained 74.75% silver, 11.56% carbon, 1.2% nitrogen, and 3.66% (m%) oxygen.

### 3.3. Transmission Electron Microscopy (TEM) Analysis

**Figure 5:**
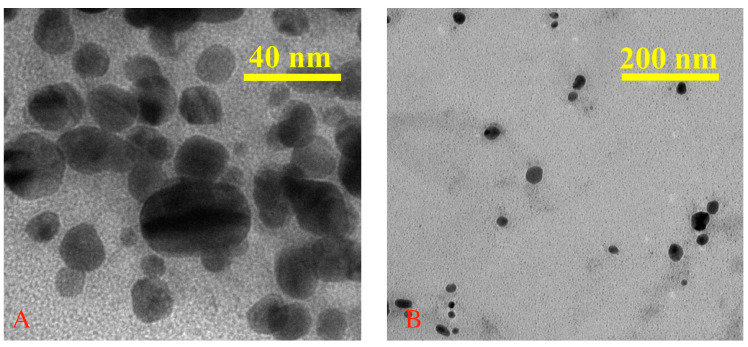
TEM micrographs of AgNPs with different magnifications (**A**,**B**).

The morphology of the nanoparticles was studied with TEM microscopy after solvent removal by evaporation at room temperature. TEM micrographs show nearly spherically shaped nanoparticles with a uniform morphology distribution without the appearance of edges, corners, or accentuation of an ovoid shape. In the dry state, the nanoparticles highlight the dimensions of the metallic Ag core, with an average diameter of 25 nm (Figure 5A). It seems that the vegetable extract effectively stabilizes the aqueous dispersion of the nanoparticles, considering that, in the TEM images at high magnification, there is no noticeable tendency for agglomeration after the removal of the solvent (Figure 5B).

### 3.4. FTIR Analysis

In order to determine the functional groups that participate in the synthesis and stabilization of AgNPs, FTIR spectra of the extract and AgNPs were recorded.

**Figure 6:**
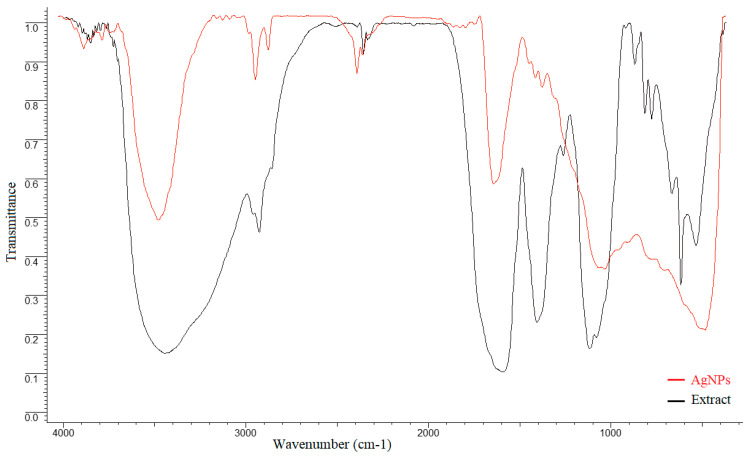
Comparative FTIR spectra of extract and AgNPs.

The extract spectrum showed significant absorbance bands at: 3442 cm^−1^, corresponding to O-H stretching intermolecular hydrogen bonding from alcohols or phenol groups; 2925 cm^−1^ and 2356 cm^−1^, representing C–H stretching of CH_3_ and CH_2_ (alkanes); 1591 cm^−1^, corresponding to C=O, C–N (amide I), and COO– stretching vibrations; 1406 cm^−1^, C–O (amide) stretching vibrations and C–C stretching vibrations of phenyl groups; COO symmetric stretching vibrations and CH_2_ bond vibrations; 1261 cm^−1^, C–O stretching vibrations of alcohols, ethers, esters, and carboxylic acids; 1116 cm^−1^, C–O and C–C stretching vibrations from carbohydrates (Figure 6).

### 3.5. Microbiological Assay

When analyzing the obtained mean CFU values in subgroups 1, it can be observed that control subgroup A1 reached the highest number of colonies, at 237.8 ± 136.2, while subgroup C1 recorded the lowest CFU value of 83.9 ± 38.2 (Table 1).

The statistical analysis showed significant differences between control subgroup A1 and subgroup C1, with a significance level of *p* = 0.012, and between control subgroup A1 and subgroup D1, with a *p* value of 0.025. In subgroups 2, the peak was reached by control subgroup A2, with a mean of 201.7 ± 112.3, and the lowest value was recorded by subgroup C2, with a value of 125.9 ± 88.1. The statistical analysis showed no significant differences between the study subgroups and the control subgroup (*p* > 0.05).

### 3.6. Vickers Hardness Test Results

In subgroups 1, the highest value was recorded by subgroup C1 (Table 2), with a mean VHN of 60.62 ± 0.32, and the samples in subgroup B1 recorded the lowest value (59.88 ± 0.4). In subgroups 2, the peak was reached by subgroup D2 (58.8 ± 0.63), while the lowest value was recorded by subgroup B2 (56.91 ± 0.59). Within both subgroups, no statistically significant difference was recorded (*p* > 0.05).

## 4. Discussion

The aim of the study was to evaluate the antibacterial efficiency and surface hardness of a microhybrid composite resin loaded with silver nanoparticles, synthesized using an *Equisetum sylvaticum* extract. Different parts of plants, such as leaves, fruit, or flowers, were used for the green synthesis of AgNPs. The advantages of this technique are its low cost, short synthesis time, and the possibility of processing at a large scale [1]. Species from the Equisetum genus are perennial plants found in the wild flora of Romania and are traditionally used as treatments against cardiovascular, neurodegenerative, or infectious diseases [18]. AgNPs were synthesized using an eco-friendly method and further characterized by UV-Vis, EDX, FTIR spectroscopy, and TEM analysis. UV-Vis comparative spectra of the AgNO_3_ extract solution and final mixture, as well as the color modification assessment at two different moments, initial and final, were performed. Initially, no absorbance peak was observed for the extract or AgNO_3_ in the 400–500 nm range, but a peak appeared at 440 nm for the mixture; thus, the modification of color and the appearance of the peak can be explained by surface plasmon resonance. The peak corresponding to the colloidal solution was large, indicating that the AgNPs solution was polydisperse. This is explained by the variety of biomolecules found in the extract with different potentials to reduce Ag+, which influences the nucleation and synthesis of AgNPs [24]. The negative Zeta potential value demonstrates that biomolecules found on the AgNPs’ surfaces are negatively charged, implying a repulsion between nanoparticles, with the colloidal solution being stable [25,26,27]. The results obtained by EDX analysis demonstrate the presence of metallic silver as well as the presence of other elements that are found in the biomolecules responsible for the capping of nanoparticles [28]. The comparative FTIR analysis showed that, in the case of AgNPs, there were some shifts or disappearances of some bands. Moreover, the same analysis highlighted that several groups of compounds are involved in the synthesis and capping of AgNPs, with the functional groups belonging to classes of compounds such as flavonoids, proteins, amino acids, sterols, carbohydrates, and phenols found in the extract [29,30,31].

The green synthesized AgNPs were added in different concentrations (0%, 0.5%, 1%, and 1.5%) by mixing in a commercial microhybrid composite resin. The obtained results were consistent with the results of other studies that found antibacterial efficacy of AgNPs against *Streptococcus mutans* in concentrations of 1% or more [32,33]. Unlike the study conducted by Jenabi et al., in which, by adding 0.5% AgNPs, the number of colonies was significantly reduced, in our study there were no changes in CFU values for the same AgNP concentration [34]. Other reports concluded that even lower concentrations of 0.025% or 0.05% AgNP can have antibacterial effects against *S. mutans*, but our study showed no significant antibacterial effect for AgNPs concentrations lower than 1% [35]. This can be explained by the different antibacterial capacities of the products according to the size, shape, method of incorporation of the particles, or size of the samples [34]. Previous studies have reported that smaller particles show a stronger bactericidal effect due to higher surface/volume ratios [35,36].

Yassaei et al. consider a 1% concentration of AgNPs to be the maximum concentration that can be added to the composite resin, since higher concentrations can present increased toxicity and alter the aesthetic properties. The same author considers that a concentration of 1% is the maximum added AgNP concentration that can present antibacterial activity and, at the same time, not affect the mechanical properties of the composite material [33]. Nonetheless, other studies have used higher concentrations of AgNPs to test the antibacterial and mechanical properties of experimental composites [37,38].

In our study, AgNPs loaded in a commercial composite resin in 1% and 1.5% concentrations showed an antibacterial effect against *Streptococcus mutans*, while the hardness of the material showed no significant changes irrespective of AgNP concentration. These conclusions agree with the results of a study conducted by Bapat et al. [39].

Silver-based nanoparticles are efficient antibacterial agents, and their effectiveness increases as the surface/volume ratio increases [33]. Previous studies have demonstrated an increased cytotoxic effect of silver oxide on pathogenic microorganisms such as gram-positive or gram-negative bacteria, affecting their multiplication potential and adherence [33,40]. Silver ions can interact with the peptidoglycan cell wall by damaging the transmembrane transport of electrons; they can interfere with bacterial proteins and the plasma membrane; or they can lyse the bacterial wall, leading to cell death [35,41,42]. An undesirable effect of silver is its increased toxicity, which limits its use in human medicine [33], but its presentation in the form of nanoparticles considerably reduces the toxicity and increases its antibacterial efficiency [41]. Therefore, in reduced quantities, silver is a non-toxic metal to animal cells, but it can be very toxic to bacterial cells [42,43].

The discovery of materials with antibacterial potential has been one of the main concerns of researchers in the past decades. Regarding silver nanoparticles, the studies carried out in this direction reported an antibacterial efficiency of composites loaded with 0.5% and 1% AgNPs on *Streptococcus mutans* after 15 days, but when evaluated after 30 days, this effect was considerably reduced [44,45]. Antibacterial property maintenance over time is necessary because composite resin restorations are long-term treatments. In our study, the experimentally obtained composite material was soaked for 7 days in a 0.01 M NaOH solution at a constant temperature of 60 °C, according to the protocol described by Prakk et al. [22]. The results showed that the antibacterial efficiency of the silver nanoparticles loaded in the composite resin at concentrations of 1% and 1.5% was considerably reduced after soaking in 0.01 M NaOH solution; these findings agree with the results of another previous study [46]. NaOH acts on the composite resin by chemical degradation, accelerating the hydrolysis process [47]. The Vickers hardness of the experimental composite resin was not affected by submersion in NaOH solution for 7 days. Other studies have reported significant changes in physical and mechanical properties after this aging procedure [47,48,49].

Nonetheless, one important limitation of the present in vitro study is the impossibility of achieving a complex environment such as that of the oral cavity. The study may also be limited by the use of Herculite XRV composite resin as a tested material due to the fact that it was launched onto the market more than 25 years ago and, as resin-based materials evolved in recent years, it has required improvements in its composition. Consequently, further in vivo or other in vitro studies that replicate the oral environment conditions considering the presence of saliva, variations of the salivary flow, enzyme activity, and thermal or pH variations are recommended to confirm the antibacterial potential and mechanical behavior of the studied material.

## 5. Conclusions

The use of 1% wt. and 1.5% wt. silver nanoparticles synthesized from *Equisetum sylvaticum* as a composite resin filler reduced the activity of *Streptococcus mutans*. Soaking of the obtained experimental composite resin in a 0.01 M NaOH solution decreased the antibacterial efficacy. The loading of a commercial microhybrid composite resin with silver nanoparticles in concentrations of 0.5% wt., 1% wt., and 1.5% wt. did not influence the surface hardness.

## Figures and Tables

**Table 1:** Mean number and standard deviation (SD) of *Streptococcus mutans* colonies (CFU) on 5% sheep blood agar.

Groups	Group A (0% wt. AgNPs)	Group B (0.5% wt. AgNPs)	Group C (1% wt. AgNPs)	Group D (1.5% wt. AgNPs)
CFU *Streptococcus mutans* -subgroups 1-	237.8 ± 136.2	145.3 ± 89.1	83.9 ± 38.2	91.7 ± 70.8
CFU *Streptococcus mutans* (samples soaked in 0.01 M NaOH solution) -subgroups 2-	201.7 ± 112.3	169.8 ± 113.4	125.9 ± 88.1	142.2 ± 105.5

**Table 2:** Mean Vickers hardness number (VHN) and standard deviation (SD) for each group recorded at surface and at base.

Groups	VHN -Subgroups 1-	VHN (Samples Soaked in 0.01 M NaOH Solution) -Subgroups 2-
A	60.14 ± 0.22	57.33 ± 0.76
B	59.88 ± 0.40	56.91 ± 0.59
C	60.62 ± 0.32	57.97 ± 0.88
D	60.48 ± 0.66	58.80 ± 0.63

## Data Availability

All the data presented in this study are available within the article.
